# Selective Attention Dynamics in Adults With Attention-Deficit/Hyperactivity Disorder: A Role for Sensory Processing Asymmetry?

**DOI:** 10.1016/j.bpsgos.2026.100715

**Published:** 2026-03-02

**Authors:** Nicolas Zink, Samantha O’Connell, Samantha Betts, Sean Noah, Austin Hilberg, Jim McCracken, Rory Reid, Susan Bookheimer, Mark S. Cohen, Stanley A. Klein, Mercedes T. Oliva, Greg V. Simpson, Agatha Lenartowicz

**Affiliations:** a Department of Psychiatry and Biobehavioral Sciences, Semel Institute for Neuroscience and Human Behavior, University of California Los Angeles, Los Angeles, California; b Department of Otolaryngology–Head and Neck Surgery, Keck School of Medicine, University of Southern California, Los Angeles, California; c Graduate Program in Neurosciences, University of Southern California, Los Angeles, California; d Department of Neuroscience, University of California Berkeley, Berkeley, California; e School of Optometry and Vision Science, University of California Berkeley, Berkeley, California; f Department of Psychiatry and Biobehavioral Sciences, David Geffen School of Medicine, University of Southern California, Los Angeles, California; g Department of Psychiatry and Biobehavioral Sciences, Neurology, Radiology, Biomedical Physics, Psychology, Bioengineering and California Nanosystems Institute, University of California Los Angeles, Los Angeles, California; h Division of Social Sciences, University of California Santa Cruz, Santa Cruz, California; i Think Now, Inc., San Francisco, California

**Keywords:** ADHD, EEG, ERP, Partial least squares, Selective attention, Subtype

## Abstract

**Background:**

Despite persisting symptomatology in adults with attention-deficit/hyperactivity disorder (ADHD), it remains elusive whether ADHD-related impairments of selective attention are domain general or specific, whether this inattention is related to earlier stimulus encoding or later stimulus-response mappings, and whether it manifests differently in ADHD presentations.

**Methods:**

Using an intermodal selective attention task, we investigated whether and when selective attention processes across competing modalities (auditory and visual) differ in adults with inattentive (*n* = 80) and combined (*n* = 31) presentations relative to unaffected adults (*n* = 37). Using multivariate statistical inference (partial least squares), we identified event-related potentials (ERPs) that differentiated conditions (attending, ignoring) and diagnostic groups.

**Results:**

Compared with controls, task performance was lower in individuals with ADHD. In both modalities, effects of attending versus ignoring were found in early (<200 ms) and late (>300 ms) processes. During the visual task, staircase-like ADHD presentation differences were found around the N2 when attending to visual and around the P3 when ignoring auditory stimuli. During the auditory task, late-processing ERPs were unaffected by ADHD. Instead, ADHD differences in sustained attention processes were observed across attended and ignored stimuli during early sensory processing (P1).

**Conclusions:**

Our data indicate that ADHD-related selective attention impairments persist into adulthood and are context dependent (dominant during visual attention) and accompanied by altered sustained attention. In the combined presentation, postsensory attention processes were more severely affected. The presence of an asymmetric selective attention impairment that favors auditory over visual attention does not support global selective attention deficits in adult ADHD. A discussion of clinical implications with real-life examples is provided.

## Plain Language Summary

Selective attention problems are common in ADHD, but it is unclear whether difficulties are specific to sensory modality and whether they arise during perception or later during response preparation. In this study, we examined these questions across different presentations of ADHD in adults. Using an EEG task requiring participants to attend to visual or auditory stimuli while ignoring the other, inattentive or combined ADHD presentations were compared with control participants. ADHD performed worse, with impairments most evident during visual attention and stronger late-stage deficits in the combined presentation. The findings indicate that ADHD-related attention problems are context dependent rather than global, informing targeted clinical strategies.

Attention-deficit/hyperactivity disorder (ADHD) is a neurodevelopmental condition marked by symptoms of inattention, hyperactivity, and impulsivity that affects approximately 10% to 11% of children (1,2) and 6% to 7% of adults globally (3) and is associated with significant cost to the quality of life of patients (4, 5, 6). For many years, ADHD was thought to be a childhood-onset disorder with limited effect on adult psychopathology (7) due to onset in childhood with diminishing symptoms over the course of adolescence (8). However, longitudinal studies have shown evidence for both persistent ADHD symptomatology (9,10), with diagnostic remission rates ranging between 30% and 70% (9,11, 12, 13), and an emerging group of adult ADHD cases with no record of childhood onset (14). Importantly, ADHD-related impairments of attention, a hallmark symptom of ADHD (15), persist at ∼70% in adults regardless of meeting ADHD diagnostic criteria (9,11, 12, 13).

A recent meta-analytic review of 93 studies encompassing 5574 adults diagnosed only with ADHD revealed deficits across all attention modalities and in processing speed and executive function (16), suggesting high overlap in cognitive (dys)functions between children and adults with ADHD. However, clinical observations suggest that adults show a different set of ADHD-related cognitive deficits in executive functioning compared with children (17) and that hyperactivity during childhood manifests more as an internal restlessness among adults (18). Moreover, the underlying mechanisms of ADHD-related impairments in adults are not well understood, warranting further efforts to understand which neurophysiological processes of attention are impaired that persist into adulthood and how ADHD-related deficits in attention are manifested differently in adults with different subtypes of ADHD.

One of the core characteristics of ADHD is impairment of sustained (19, 20, 21, 22, 23, 24) and selective attention, where attention has to be shielded (i.e., ignored) against interfering information (i.e., distractors) (25, 26, 27, 28, 29, 30, 31, 32, 33, 34, 35, 36, 37). Hierarchical attention control models such as the multiple demand network theory (38, 39, 40) assume a modular brain architecture (41, 42, 43, 44), in which attention is conceptualized as a macroconstruct that consists of anatomically segregated, functionally specialized, and hierarchically organized modules. From this perspective, the question arises whether ADHD-related impairments of attention are domain general (affecting both auditory and visual modality) or domain specific and whether inattention is related to earlier stimulus encoding or later processes are related to stimulus-response mappings.

In children with ADHD, studies have found converging evidence for differences in selective attention across task paradigms in both visual (25,28, 29, 30, 31,33, 34, 35, 36, 37) and auditory (26, 27, 28,30,32,35,36) selective attention processes. Several electroencephalography (EEG) studies have associated behavioral deficits in selective attention with alterations in early sensory processing [i.e., event-related potentials (ERPs), such as the P1 (29, 30, 31, 32,37) and N1 (31,36)], whereas others found no differences in early stimulus processing (32,33). Later higher-order cognitive processes (associated with N2 and P3 ERPs) have been consistently found to differ in childhood ADHD for both visual (28, 29, 30,33, 34, 35, 36, 37) and auditory (26,30,32,35,36) selective attention. Together, the studies on selective attention deficiencies suggest that in children with ADHD, early stimulus encoding, as well as later stimulus-response mapping processes (i.e., P3) during selective attention, are affected across sensory modalities.

However, despite difficulties with selective attention being a frequent complaint of adult patients with ADHD (45), only a few studies have investigated selective attention impairments in adults. While some found that the performance of adults with ADHD during auditory selective attention was not different from control participants’ performance (46), others found that impaired auditory selective attention performance was associated with ADHD symptoms and larger N1 modulations during early stimulus processing (47). For visual selective attention, some studies found that no ADHD-related performance impairments or early visual processing modulations in the P1 ERP were affected (48). However, other studies found that adults with ADHD and other comorbidities were less able to ignore irrelevant visual information (49). Together, these studies provide an unclear picture of selective attention deficits in adult ADHD and suggest that early stimulus processing may be differently affected in different modalities.

Therefore, the motivation for this study was to investigate whether and how early sensory and later response–related processes during selective attention are affected in adults with ADHD, how generalizable such differences are across auditory and visual modalities, and how these effects differ by diagnostic subtype (or presentations of ADHD, as defined in DSM-5). To this end, we used an experimental paradigm that requires participants to attend and ignore information in both the visual and auditory sensory modalities (50). Audiovisual attention is a domain in which electrophysiological attention effects—measured as separable differences between attended and ignored signals—have been well characterized (51, 52, 53, 54, 55, 56, 57).

Therefore, our paradigm allowed us to test whether deficits exist in either attending or ignoring processes and, furthermore, whether general ADHD-related deficits exist that impact all sensory modalities versus the alternative that such disturbances are selective to one modality.

## Methods and Materials

### Participants

We tested 148 right-handed individuals (84 women, 64 men; mean age = 25.6 ± 5.6 years). Before testing, participants completed a diagnostic assessment performed by a clinician lasting approximately 1 to 2 hours, during which the ADHD presentation was determined, either being the inattentive type (ADHD_inattentive_ group) (*n* = 80) or the combined hyperactive and inattentive type (ADHD_combined_ group) (*n* = 31). Exclusion and inclusion criteria for ADHD are identical to a previous study using the same sample (58) and are detailed in the Supplement. All participants in the ADHD group were required to meet DSM-5 diagnostic criteria for current ADHD symptomatology and confirm childhood onset of ADHD by supplemental information from medical records, parents, or other reliable sources such as school records or prior psychiatric diagnoses.

### Task

**Figure 1:**
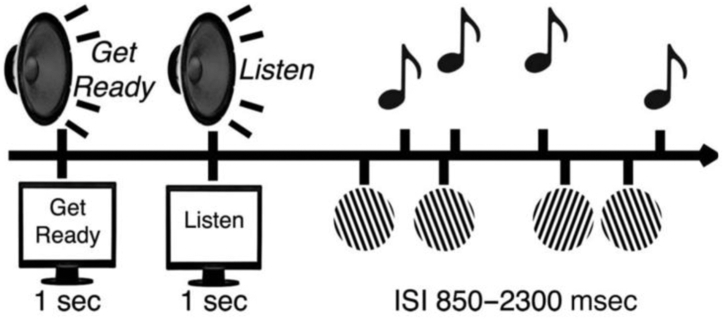
Task design as in Lenartowicz *et al.* (59, 92). Participants were presented with 2 streams of stimuli, a visual (Gabor patches) and an auditory (tones) stimulus set. They were asked to attend attention according to 3 different instructions (attend visual, attend auditory, passive) before every block. ISI, interstimulus interval.

The task and design are identical to Lenartowicz *et al.* (59). An overview of the experimental protocol is shown in Figure 1 and detailed in the Supplement. Briefly, participants were presented with 2 streams of stimuli, a visual and an auditory stimulus set, and were asked to direct their attention according to 3 different instructions (attend visual, attend auditory, and passive), varied across blocks. The attend conditions required participants to make binary decisions for each attended-modality stimulus with a forced-choice response, thus modulating attention control by attending actively to a stream of stimuli from one modality while suppressing the irrelevant stimuli from the other.

### Behavioral Data Analysis

Separate linear mixed-effects models were fit to predict effects of task modality (visual vs. auditory attention), experimental group (ADHD_inattentive_, ADHD_combined_, and control), and medication status (washout medication vs. medication naïve) on the median reaction time (RT_median_) and standard deviation of RTs (RT_SD_) and the accuracy (correct responses in percent) as well as the relative variability of RTs (coefficient of variation [RT_SD_/RT_mean_]), with age as a covariate, using unstructured covariance parameterization. The models were estimated using the restricted maximum likelihood method, which provides less biased estimates of variance components. Statistical analyses were performed using SPSS version 21.0 (IBM Corp.) statistical software package.

### EEG Analysis

EEG data acquisition and preprocessing followed previously published protocols (59) and are detailed in the Supplement. Epochs were extracted from −200 to 1200 ms after stimulus onset for all 4 stimulus classes, derived from 2 modalities embedded in 2 task contexts (visual task: attended visual stimuli and ignored auditory stimuli; auditory task: attended auditory stimuli and ignored visual stimuli). Two clusters of 5 electrodes around electrodes Pz and Oz were selected for analysis of visual ERPs (attended, ignored), and a cluster of 7 electrodes around electrode Cz was selected for analysis of auditory ERPs (attend/ignore). The rationale for selecting these electrode clusters was motivated by our earlier findings on the topographies and expected sensory effects in Lenartowicz *et al.* (59) [but also see (51, 52, 53, 54, 55, 56, 57) for similar topographies] including early stimulus processing (P50, P1, N1), visual N2, and auditory Nd—referred to as selection and processing (N2) negativities, respectively (59)—and stimulus-response mapping (P3) across the selected topographical electrode clusters. Preprocessing and statistical analysis were performed using EEGLAB (60) and Partial Least Squares software packages (61).

### Partial Least Squares on ERPs

To identify group and selective attention (attend/ignore) effects in EEG signals, we used partial least squares (PLS) (62), a multivariate technique used to identify relationships between task or group effects and multivariate dependent measures through the decomposition of their cross-block covariance matrix. The decomposition reexpresses the cross-block covariance matrix as a set of mutually orthogonal latent variables (LVs), akin to principal components in a principal component analysis. The LVs are identified in decreasing order of covariance explained, and each one comprises a 1) singular value (reflecting the proportion of explained cross-block covariance), 2) design saliences (weights within the right singular vector) representing a task contrast, and 3) electrode-time saliences (weights within the left singular vector) representing optimal spatiotemporal relation of ERP signals to the identified task contrast. Comprehensive descriptions of PLS are available in Lobaugh *et al.* (63), McIntosh and Lobaugh (61), and the Supplement. Notably, similar to ERPs, the electrodes Cz, Pz, and Oz were also within PLS clusters of electrodes that showed significant electrode-time saliences.

In the first set of PLS analyses, we identified effects on ERPs due to condition differences (attend/ignore) in the 3 groups (ADHD_inattentive_, ADHD_combined_, and control). For this, we ran separate PLS models on ERPs for auditory and visual stimuli. A second complementary analysis was run for the passive condition in both modalities (omitting the condition dimension) to identify group differences in passive stimulus ERPs. We used a randomization scheme with 1000 permutations for contrast significance estimation and 1000 bootstrap samples to estimate the stability of spatiotemporal contrast saliences. We compared task contrasts of significant (*p* ≤ .05) LVs in electrode analyses calculated by permutation tests and cross-block covariance contributions.

## Results

### Demographics and Behavior

In the entire sample, 111 were individuals diagnosed with ADHD (65 women, 46 men; mean age = 25.7 ± 5.7 years), including 80 with the inattentive (41 women, 39 men; mean age = 25.6 ± 5.5 years) and 31 with the combined (24 women, 7 men; mean age = 26 ± 6.2 years) presentation. The remaining 37 healthy participants comprised the control group (19 women, 18 men; mean age = 25.5 ± 5.6 years) (see Table 1). Sex effects were not observed. For descriptive information on current and lifetime comorbid diagnoses in experimental groups and medication status in the ADHD groups, please refer to Table 2.

**Table 1:** Demographic Data for the Experimental Groups

	Control, *n* = 37	ADHD_inattentive_, *n* = 80	ADHD_combined_, *n* = 31
Sex, Female/Male	19/18	41/39	24/7
Age, Years	25.46 (5.59)	25.56 (5.49)	25.97 (6.18)
ASRS Hyperactive	9.16 (5.62)	20.21 (5.49)	24.81 (4.45)
ASRS Inattentive	10.38 (5.69)	26.24 (4.29)	26.68 (3.99)

Values are presented as mean (SD) or *n*.

ADHD, attention-deficit/hyperactivity disorder; ASRS, Adult ADHD Self-Report Scale.

**Table 2:** Current and Lifetime Comorbid Diagnoses for the Experimental Groups and Medication for the ADHD Groups

	ADHD		Control
	Inattentive	Combined	
Current Comorbid Diagnoses			
Anxiety disorder	20.0%	16.1%	5.4%
Depressive disorder	3.8%	6.5%	0%
Depressive episode	0%	0%	0%
Dysthymia	6.3%	6.5%	0%
Panic disorder	0.0%	0%	0%
Social anxiety disorder	3.8%	0%	2.7%
Substance abuse	0%	0%	0%
Hypersexual disorder	1.3%	6.5%	0%
Lifetime Comorbid Diagnosis			
Anxiety disorder	0%	3.2%	2.7%
Depressive disorder	3.8%	0%	2.7%
Depressive episode	8.8%	12.9%	5.4%
Dysthymia	1.3%	0%	2.7%
Panic disorder	2.5%	0%	0%
Social anxiety disorder	1.3%	0%	0%
Substance abuse	3.8%	0%	0%
Hypersexual disorder	0%	0%	0%
Medication, Currently on Before Preexperimental Washout Period			
Amphetamine	13.8%	16.1%	–
Methylphenidate	7.5%	3.2%	–
Lisdexamfetamine	2.5%	3.2%	–
Dextroamphetamine	1.3%	3.2%	–
Bupropion	1.3%	0%	–

ADHD, attention-deficit/hyperactivity disorder.

**Figure 2:**
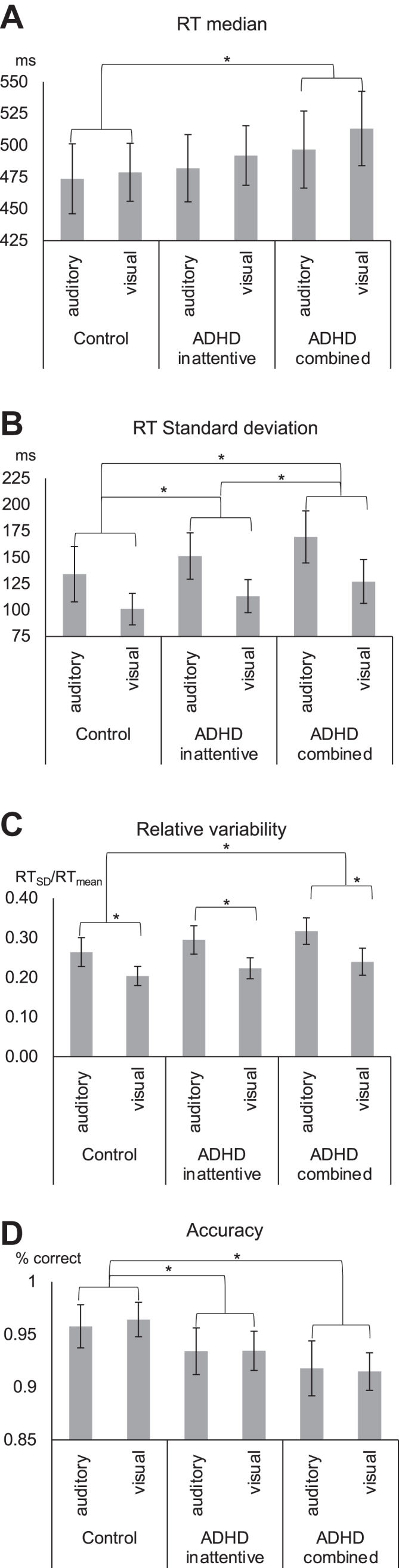
Behavioral results for each experimental group and sensory modality. Error bars denote the standard error. Asterisks denote significant pairwise (*p* < .05) post hoc *t* tests. **(A)** Median reaction times (RTs). **(B)** Standard deviation of RTs. **(C)** Relative variability of reaction. **(D)** Accuracy (% correct responses). Detailed statistics can be found in the Supplement. ADHD, attention-deficit/hyperactivity disorder.

The behavioral results of the linear mixed models (repeated measures) are summarized in Figure 2. Detailed statistics can be found in the Supplement.

In sum, the results show that the ADHD_combined_ group had the worst performance on the task, with slower, more variable, and less accurate responses than the control group across modalities (visual and auditory). However, RT variability showed a staircase pattern, revealing that the ADHD_inattentive_ group had higher variability than the healthy control group but lower variability than the ADHD_combined_ group. Thus, the ADHD_inattentive_ group showed an effect only in response variability, whereas the ADHD_combined_ group showed a group effect across all performance measures. Moreover, the data showed a slowing of response time (RT_median_ increase).

### Neurophysiological Results

#### Visual Selective Attention

**Figure 3:**
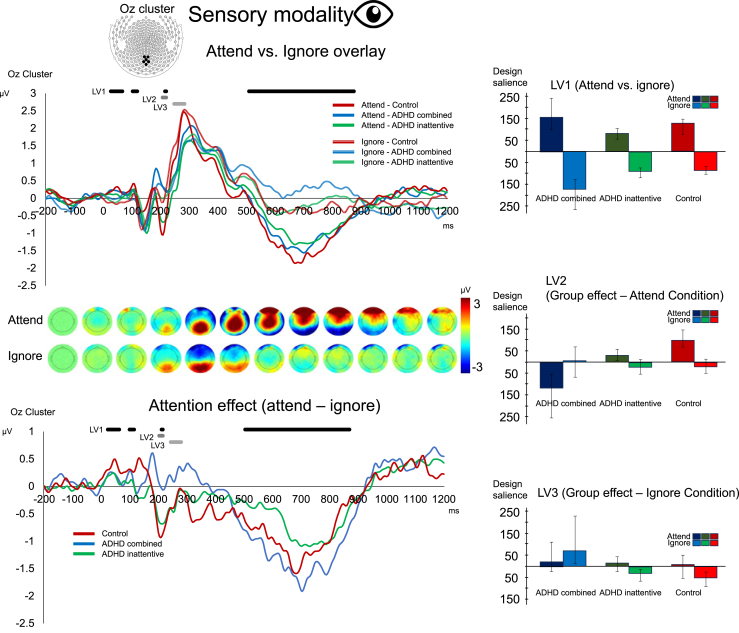
Event-related potentials (ERPs) in the Oz cluster and averaged binned topographies for the visual modality. Top: ERPs for attending (denoted in dark color shades) and ignoring (denoted in light shades) of visual stimuli. Time windows during which the latent variables (LVs) from the partial least squares analysis revealed significant (*p* ≤ .05) differences are marked in shades of gray. Bottom: ERPs of the attention effect (differences between attending and ignoring condition). Right: Bar plots with error bars (SD) for LV1–LV3 for experimental conditions (attend vs. ignore) and groups (attention-deficit/hyperactivity disorder [ADHD]_combined_, ADHD_inattentive_, control).

For visual stimulus ERPs, PLS analysis revealed 3 LVs that accounted for a significant portion of cross-block covariance (LV1 and LV2: *p* ≤ .001, LV3: *p* = .093; LV1 = 70%, LV2 = 18%, LV3 = 7%). In LV1, a main effect of selective attention (attend-ignore) was identified in time windows of early stimulus processing (50–220 ms) and around the response (500–900 ms) (see Figure 3). Across these windows, ERP amplitudes were stronger in the attend condition than in the ignore condition across all groups. In LV2, saliences identified group and ADHD subgroup differences (ADHD_combined_ < ADHD_inattentive_ < control), but only in the attend visual condition, which were significant in a time window around the N2 ERP (200 ms) (see Figure 3). Compared with the control group, the N2 was shallower for the ADHD_combined_ group relative to the ADHD_inattentive_ group, which was shallower than the control group. This is consistent with altered deployment of visual selective attention. In LV3, in the ignore visual condition in a time window around the P3 ERP (300 ms) (see Figure 3), group differences (ADHD_combined_ > ADHD_inattentive_ and control) were close to significant. In sum, the visual ERPs showed previously observed attend-ignore effects in both early (<100 ms and N2) and late (>300 ms) processes (LV1), as well as a staircase group effect (ADHD_combined_ < ADHD_inattentive_ < control) in attending to visual stimuli during the visual task, whereas ignoring of visual stimuli during the auditory task appeared unaffected.

#### Auditory Selective Attention

**Figure 4:**
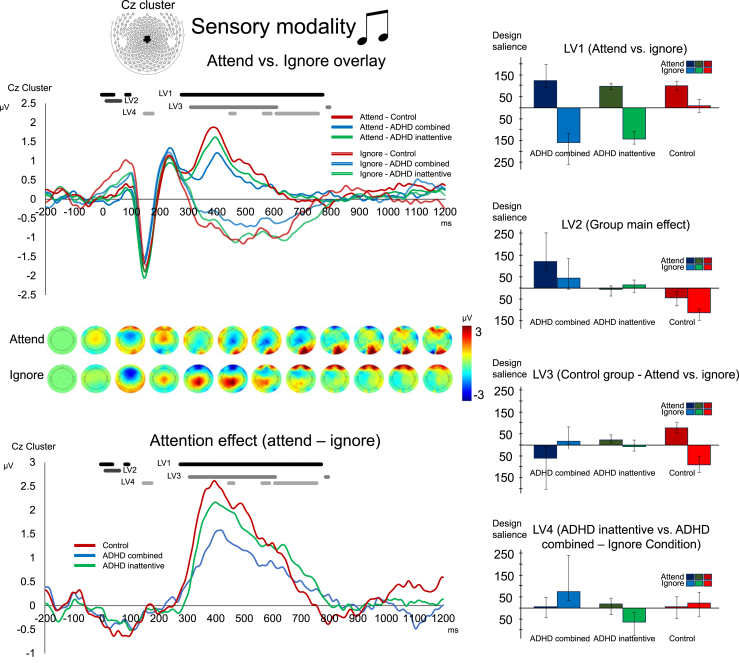
Event-related potentials (ERPs) in the Cz cluster and averaged binned topographies for the auditory modality. Top: ERPs for attending (denoted in dark color shades) and ignoring of visual stimuli (denoted in light shades). Time windows during which the latent variables (LVs) from the partial least squares analysis revealed significant (*p* ≤ .05) differences are marked in shades of gray. Bottom: ERPs of the attention effect (differences between attending and ignoring condition). Right: Bar plots with error bars (SD) for LV1–LV4 for experimental conditions (attend vs. ignore) and groups (attention-deficit/hyperactivity disorder [ADHD]_combined_, ADHD_inattentive_, control).

For auditory ERPs, PLS analysis revealed 3 significant LVs at *p* < .05 (LV1 and LV2: *p* ≤ .001, LV3: *p* = .003) as assessed by cross-block covariance accounted for (LV1 = 55%, LV2 = 22%, LV3 = 13%). LV1, as for the visual stimuli, identified a main effect of selective attention (attend − ignore), which was significant in time windows of early stimulus processing around the P1 ERP (50–100 ms) and during the processing negativity until the response (300–750 ms) (see Figure 4). Consistent with prior results, ERPs showed a stronger P3 in the attend condition than in the ignore condition, as well as a stronger early positivity in the ignore condition relative to the attend condition. LV2 was the only significant LV to identify a main effect of group (control vs. ADHD_combined_ and ADHD_inattentive_), consistent with differences during the 50 ms preceding the P1 ERP (100 ms) (see Figure 4). The variation found during the first 50 ms after stimulus onset may be attributed to background sustained attention processes. However, given its early onset, other factors, such as arousal, sensory processing quality, or baseline oscillatory activity, cannot be ruled out. The P1 amplitude (across attend and ignore stimuli) in the control group was larger than that for the ADHD_combined_ and ADHD_inattentive_ groups, across both attended and ignored auditory stimuli. LV3 identified a selective attention effect (attend vs. ignore), complementary to LV1, whereby attend versus ignore differences appeared exaggerated in the control group related to both ADHD groups. As seen in the attend-ignore difference (Figure 4), this corresponds to a greater attend-ignore effect during the poststimulus-processing stages, significant in a late negativity time window between 350 and 600 ms. In sum, auditory ERPs replicated previously observed attend-ignore effects in both early (<100 ms) and late (>300 ms) processes (LV1), but in contrast to the visual domain, attending to auditory stimuli showed no significant group differences. This result complements group effects observed for attended visual stimuli, implying a selective attention group difference only during the visual task. Additionally, a main effect of group during the first 100 ms of the stimulus for both attended and ignored auditory stimuli (LV3) suggests that sustained background processes might have contributed to differences among groups.

#### Passive Condition

**Figure 5:**
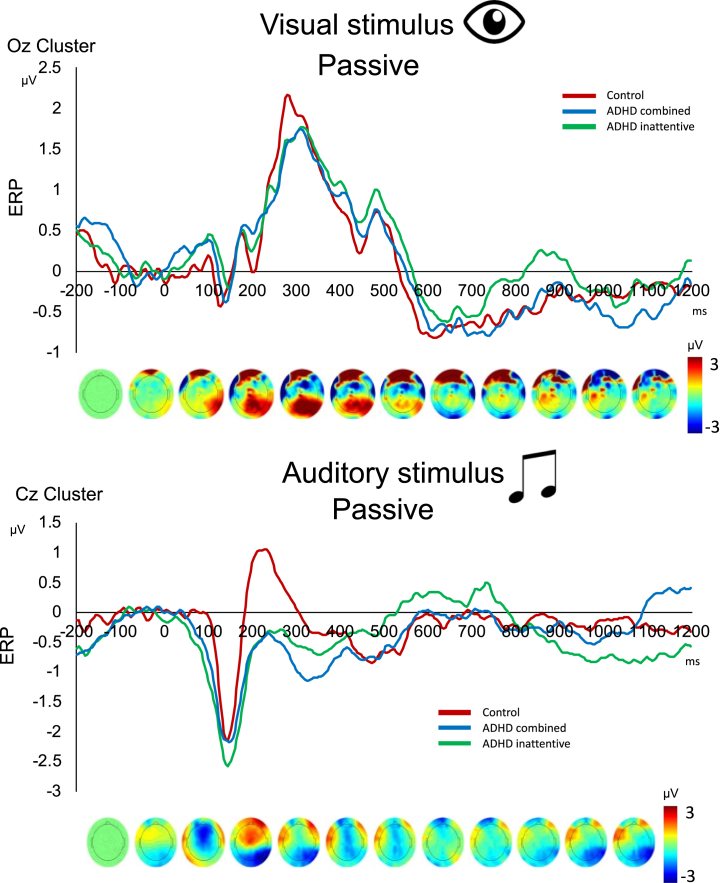
Event-related potentials (ERPs) and averaged binned topographies for the passive condition. Top: ERPs in the Oz cluster during passive visual stimulus processing. Bottom: ERPs in the Cz cluster during passive auditory stimulus processing. ADHD, attention-deficit/hyperactivity disorder.

For the passive condition, in which stimuli were processed without ignoring other distracting concurrently presented stimuli, separate PLS analyses of ERPs in visual and auditory passive stimulus processing revealed no group differences, suggesting no difference in passive sensory processing (Figure 5).

## Discussion

In this study, we used an intermodal selective attention paradigm to investigate whether and how early sensory and later nonsensory processes involved in selective attention differ in adults with ADHD. The observed timing of attention effects (attend-ignore) replicated previous reports in adults without ADHD (59). Novel to our results, we observed performance deficits in adults with ADHD. Although this study was cross-sectional and offers limited insights into onset and remission of individual ADHD-related attentional deficits, it nevertheless highlights that selective attention deficits related to ADHD may persist with greater impairment in the combined presentation. In complement, ERP analyses revealed group differences in postsensory selective attention processes, with broader and qualitatively different impairments in the combined presentation, i.e., weaker or stronger amplitudes in ERPs. Notably, these group differences were present in the visual attention condition only, suggesting an asymmetry between modalities that favors auditory over visual selective attention and thus does not support a global selective attention deficit in adult ADHD.

### Asymmetric Selective Attention Deficits?

Our results reveal that while neurophysiological differences in selective attention in adult ADHD were significant, the group effects varied as a function of the sensory modality to which attention was directed. In the visual task, group effects (control > ADHD_combined_ > ADHD_inattentive_) were found for both the attended visual stimulus in the N2 ERP and for the ignored auditory stimuli in the early N1 and later Nd ERP. This suggests that in ADHD, attending to visual targets and ignoring concurrent auditory distractors are affected in early visual selective attention processes. In the auditory task, however, group differences specific to the attended auditory stimulus were not significant. In complement, when a visual stimulus had to be ignored during the auditory task, group differences were found in the later P3 ERP that could be interpreted as beneficial to task performance because the P3 was attenuated, consistent with weaker processing of distractors. Thus, in contrast to the visual task, the neurophysiological response to stimuli during the auditory task was minimally affected by ADHD.

This result is important because it suggests that the hypothesis of a modality-general selective attention problem (or a problem occurring at single stages of processing) in ADHD in early processing is not supported. Similarly, the results are inconsistent with general deficits in only attending or only ignoring; rather, the results indicate that neurophysiological processes during selective attention are affected differently in auditory and visual task contexts. Specifically, neurophysiological processes involved in attending to auditory information were relatively unimpaired in adults with ADHD, whereas processes involved in attending to visual information were affected, with greater ERP effects in the inattentive than the combined presentation. In real-life settings, this could mean that for some adults with ADHD, focusing visual attention in noisy environments may create a greater challenge as compared with situations in which distracting visual information has to be shielded against while keeping attention focused on auditory information. This finding is intriguing as ADHD is defined regardless of sensory modality, and ADHD-related neurophysiological differences in attention have been found in both the visual (29,64, 65, 66) and auditory (27,67) domains.

Mechanistically, such asymmetry could arise from weakened deployment of visual attention, contributing to weaker attending to visual stimuli, which become less distracting when these become task irrelevant during the auditory task. Alternatively, oversensitivity to auditory stimuli could make auditory distractors more salient during the visual task while benefiting attending during the auditory task. However, the analysis of neural responses in the passive condition suggests that the latter is unlikely. In this condition, the same stimuli had to be observed in both modalities without needing to attend to or ignore another stimulus. Here, we found no evidence for general ADHD-related differences in sensory stimulus processing in either modality. Therefore, it is more likely that the pattern of results supports the hypothesis of weakened visual attention in response to visual stimuli. This view is corroborated by studies of children with ADHD, showing that attention deficiency may be present in one modality but not necessarily in the other (68) and that ADHD is more characterized by visual attention deficits (69, 70, 71), which seem to be more serious than auditory deficits (72).

Finally, we note that some studies have reported that attention deficits in children were greater in the auditory than in the visual domain (65,72, 73, 74); however, these were observed in sustained but not selective attention. In adults with ADHD, Schmidt *et al.* (75) found similar sustained attention asymmetries across modalities, with individuals with the ADHD hyperactive presentation exhibiting deficits in the auditory but not in the visual modality. However, in contrast to our results, they reported that sustained attention deficits in the ADHD_inattentive_ presentation are partially independent of the modality, which may reflect differences in sustained versus selective attention effects. In our sample, the inattentive subgroup showed greater RT variability consistent with differences in sustained attention but task accuracy and selective effects in ERPs more comparable to the control group. These data suggest that selective and sustained attention processes may make dissociable contributions to behavioral attention impairments.

### Do Subtype-/Presentation-Specific Attention-Deficit Profiles Exist in Adult ADHD?

We know that distinct neuropsychological deficits exist in children in both ADHD presentations across a range of cognitive control functions (76, 77, 78, 79, 80, 81), including sustained attention (78). Although some studies found no evidence of presentation-related differences in executive functioning profiles (71,82, 83, 84, 85, 86), including selective attention (84), growing evidence from neuroimaging studies suggests that neurobiological differences between ADHD presentations exist in children (87, 88, 89). Here, we found that selective attention neurophysiological effects and task performance showed greater group effects for the combined presentation relative to the control group than for the inattentive presentation. These results are consistent with the severity model (90), which suggests that ADHD presentations are severity variations rather than distinct etiologies. This has been supported in domains of response inhibition (76), continuous performance (78), forced attention dichotic listening (81), working memory (80,82), verbal category shifting (82), processing speed, response variability (80), and executive control (79,80).

To date, only a few studies have investigated presentation differences in the neurophysiological profile of adults with ADHD. However, a review of neural oscillation effects in ADHD (91) suggests that event-related decreases in alpha band (8–12 Hz) oscillatory activity during visual selective attention, a putative marker of visual cortex engagement, was associated with inattentive symptoms (92) and primarily found in the inattentive presentation (92,93) and less reliably in the combined presentation (93,94). These studies suggest that neurophysiological differences in ADHD presentations may exist in adults, consistent with our findings of more significant behavioral and neurophysiological differences in the combined presentation in selective attention. In contrast, Tucha *et al.* (95) found distinct profiles of attentional dysfunction in participants with ADHD versus healthy control participants but only weak differences between ADHD presentations. Given the limited data, these differences could be explained by differences in task demands (e.g., visuospatial encoding vs. selective attention). Additionally, given the clinical (90) and mechanistic (96,97) heterogeneity of the disorder, multiple causes of inattention for the different presentations of the disorder cannot be ruled out.

### Limitations

One methodological issue of this study is its cross-sectional design, which limits interpretability regarding the degree to which ADHD-related deficits in attention persist into adulthood, as ignoring and attending to visual and auditory stimuli have not been measured during childhood.

A second issue relates to the subsamples being unbalanced, such that the ADHD_inattentive_ subsample is 258% and 216% larger than the control group and ADHD_combined_ subgroup, respectively. According to Keppel (98), there is no good rule of thumb for how unequal the sample sizes need to be for heterogeneity of variance to be a problem. The PLS framework does not make hard assumptions about the joint distribution of the variables and has been shown to be robust against departures from normality (99). Although we did not consider the possibility that group size imbalance biases our PLS outcomes, a potential effect cannot be completely ruled out.

The third issue relates to the asymmetry found in ADHD-related selective attention deficits. While we did not find significant group differences in the electrophysiological signatures of the auditory task, a power issue that accounts for the null findings cannot be ruled out.

### Conclusions

Our results highlight a need for further investigations of attentional deficits in adults diagnosed with ADHD that consider the modality to which selective attention is directed, as these can be affected differently. This may facilitate the parsing of known heterogeneity in ADHD by differentiating deficits driven by asymmetric deployment of visual attention versus other causal pathways. Because most studies on ADHD-related attention impairments have been conducted with children, further studies are needed to elucidate whether ADHD-related impairments in different modalities persist or change into adulthood.

## Supplementary Material

Supplemental Methods, Results, Figures S1–S2, and Table S1

Key Resources Table
