# Supplementary material for: Selective Attention Dynamics in Adults With Attention-Deficit/Hyperactivity Disorder: A Role for Sensory Processing Asymmetry?
Source: Biol Psychiatry Glob Open Sci. 2026 Mar 2;6(3):100715. doi: 10.1016/j.bpsgos.2026.100715 (PMC13090990; doi:10.1016/j.bpsgos.2026.100715)
Supplement: Supplemental Methods, Results, Figures S1–S2, and Table S1 [file mmc1.pdf]

SUPPLEMENTARY INFORMATION

Selective Attention Dynamics in Adults With Attention-Deficit/Hyperactivity Disorder: A Role for Sensory Processing Asymmetry?

Zink *et al.*

Supplemental Material

Methods

Participants

We tested 148 right-handed individuals (84 women, age: 25.6 years ± 5.6) recruited through flyers, websites (e.g., CHADD, Children and Adults with Attention-Deficit/Hyperactivity Disorder organization), and recommendations through UCLA clinics. All participants provided written informed consent. Before testing, all subjects completed a diagnostic assessment performed by a clinician lasting about 1-2 hrs, in which the ADHD presentation was determined, either being the inattentive ($ADHD_{inattentive}$ group; N=80) or combined hyperactive and inattentive type ($ADHD_{combined}$ group; N= 31).

For the ADHD group, all subjects were required to meet DSM-5 diagnostic criteria for current ADHD symptomatology; and to confirm childhood onset of ADHD by supplemental information from medical records, parents, or other reliable sources such as school records or prior psychiatric diagnosis.

Inclusion criteria for ADHD participants included the following: (i) meeting of DSM-5 criteria for ADHD (1), presenting with clinically significant levels of impairment as assessed by structured clinical interview and the Adult ADHD Clinical Diagnostic Scale (ACDS DSM-5); (ii) Clinical Global Impression-Severity (CGI-S) as indicated by a score ≥ 4; (iii) estimated IQ equal or greater than 80 (measured by WASI-II); (iv) allowance of anxiety disorders, persistent depressive disorder (dysthymia), antisocial personality disorder, oppositional defiant disorder (ODD), conduct disorder (CD),

current nicotine abuse and substance abuse as a lifetime diagnosis as well as lifetime diagnosis of MDD that is not current, as comorbidities for the ADHD sample; (v) ability to complete required study procedures.

Exclusion criteria (assessed with the Mini International Neuropsychiatric Interviews, MINI, DSM-V) for ADHD participants were as follows: lifetime history of bipolar disorder, psychotic disorder, pervasive developmental disorder, and current major depression disorder (MDD), substance abuse, or substance dependence (except nicotine). We excluded current MDD while keeping Persistent Depressive Disorder (PDD) because MDD involves being in episodes, while PDD is often considered a syndrome marked by lower levels of symptom severity without a clear episodic course. We felt that current MDD would interfere too much with their cognitive testing, thereby confounding the study data and assessment.

Exclusion criteria for ADHD and neurotypical participants included the following: (i) history of childhood neurodevelopmental disorder other than ADHD (e.g., autism, dyslexia); (ii) history of a general medical condition requiring chronic use of medication with CNS effects on cognitive performance; (iii) history of seizure disorder, brain tumor, other major neurological disorder, or head injury resulting in loss of consciousness; (iv) history of serious oxygen deprivation; (v) current psychopathology requiring ongoing treatment with antipsychotic medications, mood stabilizers, benzodiazepines, or anticonvulsants; (vi) current untreated psychopathology rated as greater in severity than ADHD per se; and (vii) current treatment with guanfacine (because of the unacceptable risks of rapid withdrawal).

All subjects completed the Adult ADHD Self-Report Scale (2) (ASRS v1.1), an 18-item self-report questionnaire designed to assess Attention Deficit Hyperactivity Disorder (ADHD) symptoms in adults, which was used to inform clinician judgment in assessing ADHD and to determine additional exclusion criteria. This study was approved by the local investigational review board.

Task

The task and design are identical to that used by Lenartowicz et al. (3). An overview of the experimental protocol is shown in Figure 1. Briefly, participants were presented with two streams of stimuli, a visual and an auditory stimulus set, and were asked to direct their attention according to three different instructions (attend visual, attend auditory, passive), varied across blocks. The attend conditions required binary decisions for each attended-modality stimulus with a forced-choice response, thus modulating attention control by attending actively to a stream of stimuli from one modality while suppressing the irrelevant stimuli from the other. The attend visual condition required attending and responding to the visual stimuli (circular Gabor patches) and ignoring the auditory tones. Participants had to decide if the visual stimuli were oriented vertically (standard orientation) or tilted diagonally (off-vertical). These sinusoidal gratings were 5.7 in. in diameter, with a spatial frequency of 1.36 cycles/deg alternating between gray and white, and were presented centrally on a gray background of a 20-in. PC monitor (1680 × 1050 resolution, refresh rate 60 Hz, Dell, Round Rock, TX). The attend auditory condition required attending and responding to the auditory stimuli (binaurally presented tones) while ignoring the visual stimuli. Participants had to decide if the auditory stimuli were at a standard frequency of 700 Hz or if they were higher/lower. We included two nonstandard stimuli in each domain

to curb the effects of neural habituation and boredom. Nonstandard stimuli (off-vertical visual and non-700-Hz auditory stimuli) were adapted to each participant using a 3-up/1-down staircase protocol ((4); k = 0.3, delta ratio = .7393) to ensure that stimuli (e.g., vertical vs. off-vertical left/right and 700 Hz vs. higher/ lower) could be discriminated with at least 83% accuracy. For the auditory stimuli, an average off-vertical rotation of 12° counterclockwise (SE = 3.9°) and 11° clockwise (SE = 3.7°) relative to vertical was identified. For the auditory stimuli, a 598 Hz (SE = 23 Hz) and 803 Hz (SE = 25 Hz) tone relative to 700 Hz was identified.

Finally, attending conditions alternated with blocks of a passive control condition, in which participants received both streams of stimuli but were instructed to neither attend nor respond to any stimuli while maintaining gaze fixation at the center of the screen. These conditions were included to provide a neutral reference, quantifying neural responses to the stimulus streams in the absence of a cognitive task.

Participants performed a total of four blocks. Each block comprised two 40-sec "mini-blocks" of each of the three task conditions (attend auditory, attend visual, passive), ordered randomly across blocks and participants. Each miniblock comprised 25 auditory and 25 visual stimuli, resulting in eight miniblocks or 200 trials per condition. A single-word audiovisual instruction (presented both on screen as a written word and spoken aloud through the speakers) was presented before for 1 sec each miniblock, followed by a 3-sec gap, and then by the 40-sec mini- block and a 17-sec break period. Within each mini-block, only one of the two nonstandard stimuli, randomly selected, was presented along with the standard stimuli (i.e., vertical and 700 Hz). Standard and nonstandard stimuli occurred with equal probability. All stimuli had durations of 100

msec. To minimize expectation-based strategies and thus maximize the interfering effect of the ignored stream on the attended stream, stimulus onsets of both visual and auditory stimuli were independently jittered with an inter-stimulus interval (ISI) ranging from 700 to 2000 msec randomly sampled from a uniform distribution. ISIs were adjusted so no two stimuli were presented within 350 msec of each other to prevent multisensory effects on perception (5).

Participants responded using the index and middle fingers of the right hand and the “<” and “>” keys of a QWERTY keyboard. Finger assignments to responses were varied randomly across participants. The stimuli, auditory pure tones (sampled at 22,050 Hz, 10 msec ramp up and down) and visual gratings, were produced in Matlab (Mathworks, Natick, MA; v7.10). The experiment was programmed in PsychToolbox, running on an Apple MacBook Pro computer (Apple Computer, Cupertino, CA; OS 10.6.8).

EEG Data Acquisition

EEG data was collected using a 256-electrode HydroCel Geodesic Sensor Net (EGI, Eugene, OR), digitized using a Net Amps 300 amplifier (10,000 Hz anti-aliasing filter; common-mode rejection 90 dB; input impedance 200 MΩ) and sampled at 250 Hz. We kept electrode impedances below 50 kΩ. During the recording, electrodes were referenced to the vertex electrode but were re-referenced offline to a common average. We used NetStation software (v.4.4; EGI) to control the acquisition.

EEG Preprocessing

We preprocessed and analyzed the data using the EEGLAB toolbox (6); v. 10.2.5.5). We first applied a high-pass filter (>1 Hz) and inspected the data visually for noisy channels, which, when present, were replaced using spherical interpolation. The choice for setting the high-pass filter to 1 Hz was to smooth ERPs and increase the signal-to-noise ratio by removing slow drift processes unrelated to selective attention. Though such a filter is known to impact peak shape, the present analysis was focused on previously published effects of attending and ignoring on visuo-auditory ERPs, and for direct comparison with prior findings (3) we retain the same processing parameters. As such, the ERP waveforms were evaluated against known priors to ensure any such distortion is negligible.

For each subjects' dataset, to isolate artifacts, we ran an extended INFOMAX temporal ICA (7). We identified independent components visually (ICs) that accounted for eye movements, muscle, and high-frequency noise, which we removed from the raw data. We then extracted data epochs from 100 msec before to 500 msec after stimulus onset. We inspected these visually for any remaining high-amplitude noise; if present, we removed these epochs from further analysis. Across participants, this procedure resulted in an average of 185 epochs (range: 180-200; SD = 9.5) (92.5%) remaining in each condition. We defined the baseline as the 100-msec prestimulus period and subtracted the mean of this period from the poststimulus interval.

Partial least squares on event-related potentials

To identify group and selective attention (attend/ignore) effects in EEG signals we used Partial Least Squares (PLS) (8). PLS is a multivariate technique that can be used

to identify relationships between task or group effects and multivariate dependent measures through the decomposition of their cross-block covariance matrix. It has been successfully deployed in neuroimaging studies, including ERPs (9–13), in which multivariate sampling of the brain signals (e.g., via multiple electrodes or voxels in fMRI) creates a risk of false alarms and overly strict multiple correction requirements when applying univariate statistics. One of the primary applications of PLS in functional neuroimaging studies, of relevance to the current study, is to identify major experimental effects that consistently differentiate neuroimaging signal intensity across conditions and groups.

Comprehensive descriptions of PLS are available in Lobaugh et al. (2001) and McIntosh and Lonaugh (2004). Briefly, the data matrix in PLS is organized such that rows correspond to subjects within conditions & groups, and columns correspond to time points within electrodes. Each row of the matrix comprises the across-subject mean for each group and condition and in columns is the ERP time series concatenated horizontally across electrodes. This data matrix is then mean-centered column-wise with respect to the grand average. The mean-centered matrix is decomposed using singular value decomposition (SVD) to generate a set of mutually orthogonal latent variables (LVs), which are akin to principal components in a principal component analysis. The LVs are identified in decreasing order of covariance explained and each one comprises a (1) singular value (reflecting the proportion of cross-block covariance that is explained), (2) design saliences (weights within the right singular vector) representing a task contrast and (3) electrode-time saliences (weights within the left singular vector) representing the optimal spatiotemporal relation of the ERP signals to the identified task contrast.

It is possible to assess the relative strength of each LV by calculating the proportion of cross-block covariance that is explained. This can be achieved by dividing the square of the singular value of a given LV by the sum of the squared singular values for all LVs. It is important to note that this metric should not be confused with the percentage of total variance accounted for, as it only relates to the covariance between measured brain activity and experimental design, and thus is influenced by the design's parameterization.

Resampling procedures are used to support statistical inference in PLS. In the first step, permutation tests are used to assess the significance of the singular value for each LV. This involves randomly reassigning conditions within subjects. In the second step, bootstrap resampling of subjects within conditions is used to test the stability of the electrode saliences of the significant LVs across subjects. The ratio of salience to the bootstrap standard error approximates a z-score and is used to quantify effect size. By setting thresholds for bootstrap ratios, we can identify the spatiotemporal pattern of electrodes and time points that show non-zero LV saliences, indicating that they express the corresponding design contrast. In summary, grand mean deviation PLS analysis identifies main data-driven task effects and corresponding spatiotemporal signatures that are stable across subjects.

In the first set of PLS analyses, we identify effects due to condition differences (attended versus ignored) within the three groups ($ADHD_{inattentive}$ and $ADHD_{combined}$ and Control). For this, we ran separate PLS models for auditory and visual stimuli. A second complementary analysis was run separately for the passive condition in both

modalities (omitting the condition dimension) to identify group differences in passive stimulus ERPs. We used a randomization scheme with 1000 permutations for contrast significance estimation and 1000 bootstrap samples to estimate the stability of spatiotemporal contrast saliences. We compared task contrasts of significant (p <= 0.05) latent variables in electrode analyses calculated by the permutation tests and cross-block covariance contributions.

Results

For all linear mixed-effects models, except for median reaction times (F, 1, 286) = 8.356, p = .004, medication status did not show a significant effect (all F <= .599, p >= .439) or interaction (all F <= 1.173, p >= .280). Similarly, except for median reaction times (F, 1, 214) = 8.236, p = .005), no effects (including interactons) of medication status were found when the Control group was excluded (ADHD presentations only; all F <= 1.106, p >= .294), and without group as a fixed effect (ADHD group only without presentations; all F <= 1.348, p >= . 247). Further, including age as a covariate did not decrease the residual parameter estimates of the models, indicating no significant effect on the behavioral outcomes.

For median reaction times (RT), the overall model was significant (F, 1, 286) = 15903.189, p < .001), and an effect of group was found, (F, 2, 286) = 7.864, p < .001). Pairwise t-tests showed that compared to the Control group (476 ms ± 47), the ADHD$_{combined}$ group (505 ms ± 54) had significantly increased RTs across both auditory and visual modality [t(66) = -2.34, p = .023].]. In contrast, differences between Control and ADHD$_{inattentive}$ (487 ms ± 44) and ADHD$_{inattentive}$ versus ADHD$_{combined}$ were not significant (all t <= 1.74, p >= .214). However, neither stimulus modality (F, 1, 286) =

.2.894, *p* = .09) nor group * stimulus modality interaction (F, 2, 286) = .111, *p* = .895) showed significant effects. Levene's test for equality of variances indicated that the variances were homogeneous for visual and auditory modality [F(2, 145) = 1.4, *p* = .25; F(2, 145) = .37, *p* = .693].

Regarding the variability of RTs (RT_{std}), the overall model was significant (F, 1, 286) = 1889.736, *p* < .001). A group effect was found (F, 2, 286) = 9.530, p < .001), with posthoc pairwise t-test showing that across modalities, both $ADHD_{inattentive}$ [132 ms ± 33; t(115) = -2.15, *p* = .033] and $ADHD_{combined}$ group [148 ms ± 41; t(66) = -3.21, *p* = .002] had larger RT_{std} than the Control group (118 ms ± 38). Further, a significant effect of stimulus modality was found (F, 1, 286) = 36.797, p < .001). Pairwise t-tests revealed a larger RT_{std} in the auditory (151 ms ± 31) compared to the visual modality (113 ms ± 31). The group * stimulus modality interaction (F, 2, 286) = .272, p = .762), however, was not significant. Levene's test for equality of variances indicated that the variances were homogeneous for visual and auditory modality [F(2, 145) = 2.22, *p* = .112; F(2, 145) = .9, *p* = .409].

For the relative variability of reaction times (coefficient of variation: RT_{std}/RT_{mean}) the overall model was significant (F, 1, 286) = 2997.597, *p* < .001). Further, a significant effect of group was found (F, 2, 286) = 7.077, *p* < .001). Posthoc pairwise t-test showed that across modalities, $ADHD_{inattentive}$ [.259 ± .006; t(115) = -1.91, *p* = .058] did not differ in relative variability from the Control group (.234 ± .009), whereas the $ADHD_{combined}$ group (.278 ± .01) had significantly larger relative variability than the Control group [t(66) = -2.53, *p* = .014].

Moreover, a significant effect of stimulus modality was found (F, 1, 286) = 53.777, *p* < .001), with larger relative variability in the auditory modality (.292 ms ± .006) than in the visual modality (.222 ± .005). However, the group * stimulus modality interaction (F, 2, 286) = .425, *p* = .654) did not show a significant effect. Levene's test for equality of variances indicated that the variances of the relative variability of reaction times were homogeneous in the visual and auditory modality [F(2, 145) = .743, *p* = .477; F(2, 145) = .131, *p* = .878].

For accuracy (percent correct responses), the overall linear mixed model was significant (F, 1, 286) = 94710.856, *p* < .001). Further, a significant effect of group was found, (F, 2, 286) = 19.385, p < .001) with posthoc pairwise t-test showing that across modalities, both $ADHD_{inattentive}$ [93.4% ± 4; t(115) = -2.15, *p* = .001] and $ADHD_{combined}$ groups [91.6 % ± 4; t(66) = -3.21, *p* < .001] had lower accuracy than the Control group (96.1% ± 4). $ADHD_{inattentive}$ and $ADHD_{combined}$ group, however, did not differ in accuracy [t(109) = -2.16, *p* = .067]. However, neither stimulus modality (F, 1, 286) = .204, p = .652) nor group * stimulus modality interaction (F, 2, 286) = .066, p = .936) showed a significant effect. Levene's test for equality of variances indicated that the variances were homogeneous for the visual modality [F(2, 145) = 1.56, *p* = .214] but not for the auditory modality [F(2, 145) = 3.16, *p* = .045].

		Normality test					
		Kolmogorov-Smirnov			Shapiro-Wilk		
		Statistic	df	Significance	Statistic	df	Significance
Accuracy (correct responses)	Auditory	.152	148	.000	0.883	148	.000
	Visual	.151	148	.000	0.877	148	.000
Reaction time median	Auditory	.059	148	.200	0.989	148	.32
	Visual	.068	148	.092	0.988	148	.253
Reaction time standard deviation	Auditory	.072	148	.055	0.944	148	.000
	Visual	.11	148	.000	0.948	148	.000
Reaction time coefficient of variation	Auditory	.074	148	.046	0.942	148	.000
	Visual	.095	148	.002	0.942	148	.000

Table S1. Normality tests for the behavioral metrics.

Figure 1S

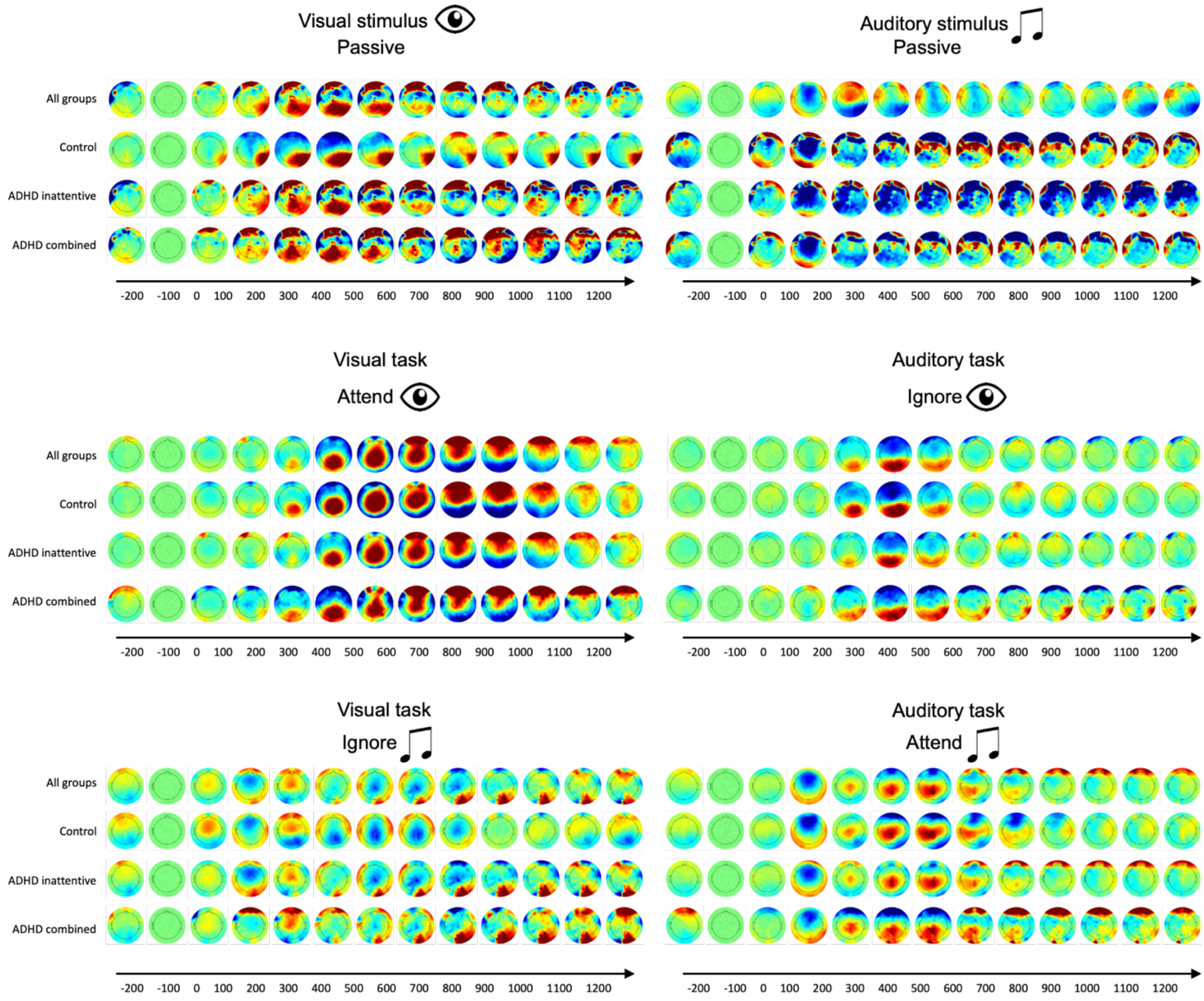

Figure 1S. Averaged topography bins for all experimental conditions and groups.

Figure 2S

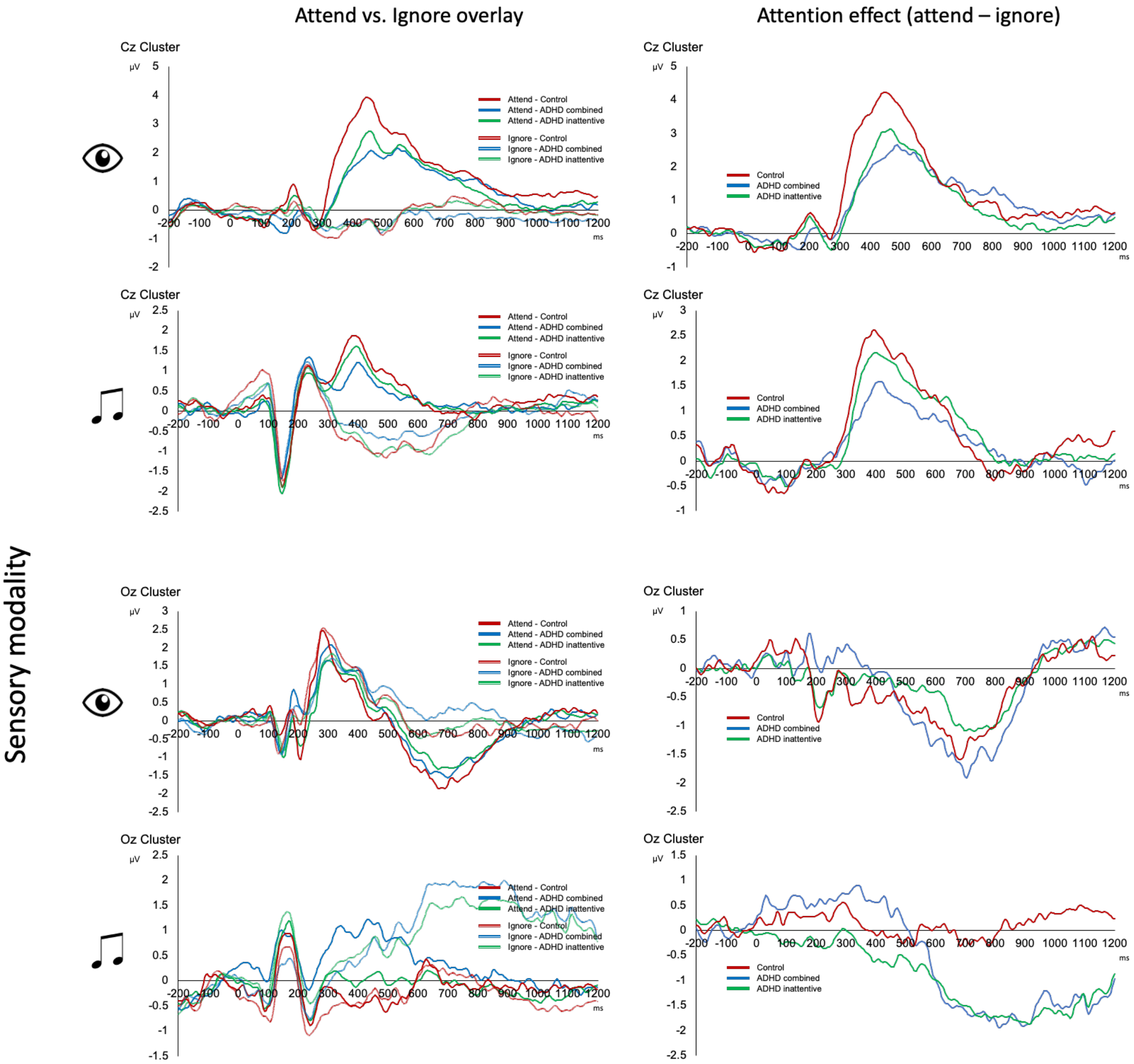

Figure 2S. Left column. Event-related potentials (ERPs) for attended (bold) vs. ignore (dashed) experimental conditions, sensory modality (visual vs. auditory), group (ADHD combined, ADHD inattentive, Control), and electrode cluster (Cz cluster vs. Oz cluster). Right column. ERPs for the attention effect (attend minus ignore).

References

1. Association AP (2013): *Diagnostic and Statistical Manual of Mental Disorders (DSM-5®)*. American Psychiatric Pub.
2. Kessler RC, Adler L, Ames M, Demler O, Faraone S, Hiripi E, *et al.* (2005): The World Health Organization adult ADHD self-report scale (ASRS): a short screening scale for use in the general population. *Psychological Medicine* 35: 245–256.

3. Lenartowicz A, Simpson GV, Haber CM, Cohen MS (2014): Neurophysiological Signals of Ignoring and Attending Are Separable and Related to Performance during Sustained Intersensory Attention. *J Cogn Neurosci* 26: 2055–2069.
4. García-Pérez MA (1998): Forced-choice staircases with fixed step sizes: asymptotic and small-sample properties. *Vision Research* 38: 1861–1881.
5. Spence C, Squire S (2003): Multisensory Integration: Maintaining the Perception of Synchrony. *Current Biology* 13: R519–R521.
6. Delorme A, Makeig S (2004): EEGLAB: an open source toolbox for analysis of single-trial EEG dynamics including independent component analysis. *Journal of neuroscience methods* 134: 9–21.
7. Lee T-W, Girolami M, Sejnowski TJ (1999): Independent component analysis using an extended infomax algorithm for mixed subgaussian and supergaussian sources. *Neural computation* 11: 417–441.
8. McIntosh AR, Bookstein FL, Haxby JV, Grady CL (1996): Spatial pattern analysis of functional brain images using partial least squares. *Neuroimage* 3: 143–157.
9. Hay JF, Kane KA, West R, Alain C (2002): Event-related neural activity associated with habit and recollection. *Neuropsychologia* 40: 260–270.
10. Itier RJ, Taylor MJ, Lobaugh NJ (2004): Spatiotemporal analysis of event-related potentials to upright, inverted, and contrast-reversed faces: Effects on encoding and recognition. *Psychophysiology* 41: 643–653.
11. Lenartowicz A, Escobedo-Quiroz R, Cohen JD (2010): Updating of context in working memory: An event-related potential study. *Cognitive, Affective, & Behavioral Neuroscience* 10: 298–315.

12. Lobaugh NJ, West R, McIntosh AR (2001): Spatiotemporal analysis of experimental differences in event-related potential data with partial least squares. *Psychophysiology* 38: 517–530.

13. McIntosh AR, Lobaugh NJ (2004): Partial least squares analysis of neuroimaging data: applications and advances. *Neuroimage* 23: S250–S263.
